# A Developmental Social Neuroscience Perspective on Infant Autism Interventions

**DOI:** 10.1146/annurev-devpsych-120621-042753

**Published:** 2023-12

**Authors:** Geraldine Dawson, Amber D. Rieder, Mark H. Johnson

**Affiliations:** 1 Duke Center for Autism and Brain Development, Department of Psychiatry and Behavioral Sciences, Duke University, Durham, North Carolina, United States; 2 Duke Global Health Institute, Duke University, Durham, North Carolina, United States; 3 Department of Psychology, University of Cambridge, Cambridge, United Kingdom; 4 Centre for Brain and Cognitive Development, Birkbeck, University of London, London, United Kingdom

**Keywords:** autism, infant, brain, intervention, developmental neuroscience

## Abstract

Research on early biomarkers and behavioral precursors of autism has led to interventions initiated during the infant period that could potentially change the course of infant brain and behavioral development in autism. This article integrates neuroscience and clinical perspectives to explore how knowledge of infant brain and behavioral development can inform the design of infant autism interventions. Focusing on infants ≤12 months, we review studies on behavioral precursors of autism and their neural correlates and clinical trials evaluating the efficacy of infant autism interventions. We then consider how contemporary developmental social neuroscience theories of autism can shed light on the therapeutic strategies used in infant autism interventions and offer a new perspective that emphasizes improving child outcome and well-being by enhancing infant–environment fit. Finally, we offer recommendations for future research that incorporates brain-based measures to inform individualized approaches to intervention and discuss ethical issues raised by infant autism interventions. Readers are referred to Supplemental Table 1 for a glossary of terms used in this article.

## INTRODUCTION

As research on early autism biomarkers and behavioral precursors allows for identification of infants with a higher likelihood of an autism diagnosis, infant autism interventions that could potentially alter the course of brain and behavioral development and lead to more positive outcomes are being developed. In this article, we integrate contemporary social neuroscience and clinical perspectives to explore how our knowledge of brain and behavioral development in infants later diagnosed with autism can inform the design of infant interventions and lead to testable hypotheses about how such interventions influence underlying neural systems. We focus on interventions with infants ≤12 months of age who are exhibiting autism-related behaviors and/or have a higher likelihood of a diagnosis of autism due to genetic factors (i.e., infant siblings of children diagnosed with autism; hereafter referred to as infant siblings).

We begin by reviewing research on infant behavioral precursors of autism and their neural correlates. This research has identified a set of core domains related to attention, social engagement, communication, and motor skills in which early differences have been identified in infants who are later diagnosed with autism. Developmental science has documented the important role of these domains in typical early social, language, and cognitive development (Tomasello & Farrar 1986, Warlaumont et al. 2014). Thus, differences in these domains can potentially have cascading consequences affecting the later well-being and quality of life of the child. Similarly, taking a holistic view on infant development, we describe several early physical health conditions associated with autism and discuss their potential impact on subsequent brain and behavioral development.

Next, we review findings from studies that evaluated the efficacy of infant autism interventions. We then offer a developmental social neuroscience perspective on infant autism interventions by exploring how current hypotheses regarding the neural basis of autism can shed light on therapeutic strategies used in infant autism interventions. This leads to a new perspective on infant interventions and assessing outcomes that emphasizes enhancing infant–environment fit with a goal of facilitating meaningful and sustained changes in those skills that improve the child’s quality of life and well-being and nurture autistic strengths. Finally, we offer recommendations for future intervention research that incorporates brain-based measures to better inform individualized approaches to intervention and discuss ethical issues raised by infant autism interventions.

## BEHAVIORAL PRECURSORS AND THEIR NEURAL CORRELATES IN THE FIRST YEAR

Research on behavioral precursors of autism began with studies of home videos of infants later diagnosed with autism (Adrien et al. 1993, Osterling & Dawson 1994). These studies documented differences in pointing, social gaze, orienting to name, smiling, facial expressions, motility, and attention by 1 year of age. One of the first longitudinal case studies of an infant later diagnosed with autism was based on a neurologist’s detailed medical chart notes from birth through 1 year, when the infant was evaluated for autism (Dawson et al. 2000). During the first 6 months after birth, this infant exhibited hypersensitivity to touch and reduced oral motor control, was socially responsive, vocalized, and smiled at others. During the second half of the first year, hypersensitivity, poor motor control, and hypotonia continued, and motor stereotypies and reductions in social gaze, social play, and imitative vocalizations were observed. In the mid-2000s, prospective studies of infant siblings began, which further shed light on the development of infants with later autism (Zwaigenbaum et al. 2007). These studies have identified four core domains—attentional flexibility, social attention and engagement, prelinguistic development, and sensory and motor differences—in which behavioral precursors of autism are consistently documented during infancy, representing focus areas for intervention. We describe these domains and their neural correlates next.

### Attentional Flexibility

In the first months after birth, as visual acuity increases, interaction with the visual environment via movements of the eyes and head feeds forward into higher-level brain regions (frontoparietal orienting networks), facilitating the development of top-down modulation of visual attention (Amso & Scerif 2015). Between 4 and 6 months, visual-orienting mechanisms become more sophisticated, supporting the ability to suppress competing stimuli during attention. The development of attentional flexibility is reflected in an infant’s ability to engage and sustain their attention to a toy or person and readily shift their focus of attention when a new object is introduced, alternating attention between the toy and person.

Attentional flexibility has been studied by the gap-overlap task, which measures saccade latencies to a target either in the face of a competing central stimulus (overlap condition) or not (gap and baseline conditions). Reaction times to orient to the target are typically slower in the overlap condition as prior disengagement from the competing central stimulus is required. There is mixed evidence whether difficulties in attentional flexibility are present by 6–7 months in infants later diagnosed with autism. One study (Elison et al. 2013a) found that 7-month-old infant siblings with later autism had longer orienting latencies in both the overlap and gap conditions compared with infant siblings without autism and infants with no family history of autism (lower likelihood infants). Another study found that 6-month-old infant siblings with later autism did not exhibit delayed visual orienting and disengagement (Bryson et al. 2018). While latencies to disengage attention in the overlap condition decrease in neurotypical development, infants later diagnosed with autism do not show this gain in flexible control of visual attention, and slower attention disengagement is reliably demonstrated by 12–14 months (Bryson et al. 2018, Elsabbagh et al. 2013). An association between visual-orienting latencies and white matter organization along corticospinal pathways, splenium, and corpus callosum has been reported for 7-month-old infants without an autism diagnosis; this association was not found for infants with later autism (Elison et al. 2013a).

A longitudinal study of infant siblings and lower likelihood infants from 6–36 months examined infants’ ability to engage, disengage, and sustain their attention during play with toys and an adult (Sacrey et al. 2013). At 12 months, infants with later autism exhibited prolonged staring at a toy after grasping it and slower attention disengagement from the toy. This made it less likely that the infant would visually explore their environment and look at the adult after grasping an object (i.e., shared attention). Consistent with a more focal attention style, superior visual search has been noted in 9-month-old siblings with later autism (Cheung et al. 2018), suggesting that visual distractors may have less influence on attention in these infants.

### Social Attention and Social Engagement

Social attention, pertaining to increased salience of and attention to faces, eyes, and voices, is reduced by 6–8 months of age in infants later diagnosed with autism (Chawarska et al. 2013, Jones & Klin 2013, Jones et al. 2016). Reduced social attention and gaze following have been hypothesized to contribute to early differences in face processing in infants later diagnosed with autism (Dawson et al. 2005, Jones et al. 2016, Mundy & Bullen 2021). In a longitudinal study of infant siblings during social interaction, infants with later autism showed declining trajectories of attention to an adult’s face, looking less to the adult’s face than neurotypical infants by 12 months (Ozonoff et al. 2010). Another study reported reduced attention to faces during social interaction in 6-, 9-, and 12-month-old infants later diagnosed with autism. This difference was most apparent when interactions involved speaking and tickling versus singing and playing with a toy (Macari et al. 2021). Speaking and tickling are less predictable and potentially more arousing. Consistent with this, in a study of toddlers with versus without a sibling with autism, toddlers who were later diagnosed with autism looked at faces more when the interaction was more predictable (Vernetti et al. 2018). These findings and earlier similar results suggest that social attention in infants and toddlers diagnosed with autism increases when interactions are more predictable and immediately contingent on the child’s behavior (Lewy & Dawson 1992). In addition to reduced attention to faces, reduced responding to name is observed by 9 months and strengthens as a predictor of autism during toddlerhood (Miller et al. 2017), becoming specific to autism after one year (Hatch et al. 2021).

Infants later diagnosed with autism exhibit diminished social engagement during social interaction, including reduced positive affect, by 6 months of age (Garon et al. 2022). By 9 months, reduced eye gaze, facial expressions, gestures, and vocalizations during interaction are observed (Bradshaw et al. 2021).

Neurophysiological measures, including event-related potentials (ERPs), electroencephalography (EEG) spectral power densities and microstates, and near-infrared spectroscopy, have identified reduced responsiveness to faces in infants later diagnosed with autism (Gui et al. 2021a, 2021b; van Noordt et al. 2022), evident by 4–6 months of age (Jones et al. 2016, Lloyd-Fox et al. 2018). These findings point to early differences in activity of the fusiform face area in the inferior temporal lobe and related brain regions [e.g., amygdala, superior temporal sulcus (STS), temporoparietal junction].

### Prelinguistic Development: Vocalizations, Gestures, and Joint Attention

Infants typically transition from nonsyllabic to syllabic vocalizations by 7 months and increasingly use canonical vocalizations (speech-like consonant–vowel combinations) over the next several months. Nine-to-twelve-month-old infants later diagnosed with autism produce fewer canonical or speech-like vocalizations and more frequent noncanonical or nonspeech-like vocalizations (Plate et al. 2022). Caregivers respond more frequently to canonical vocalizations, which shapes babbling development (Warlaumont et al. 2014). Infant siblings with later autism are less likely to socially direct their vocalizations (Garrido et al. 2017).

On the basis of functional magnetic resonance imaging (fMRI), a lack of right hemisphere specialization of the temporal and medial frontal regions for human voice processing was found in infant siblings (Blasi et al. 2015). In another fMRI study, hyperconnectivity between auditory and somatosensory regions involved in language was found in 9-month-old infant siblings (Liu et al. 2020). EEG power and nonlinear EEG features, collected at 6 and 12 months while listening to phonemes, predicted later autism in infant siblings (Peck et al. 2021).

The use of gestures and joint attention is also delayed in infants later diagnosed with autism. Gaze following, a precursor of joint attention, is reduced in 8-month-old infant siblings (Bussu et al. 2021). From 8 to 14 months, infants with later autism show slower growth in gestures and gesture–vocal coordination (Bradshaw et al. 2021). At 12 months, infant siblings with later autism produce fewer gestures, especially gestures integrated with speech, and gesture use is predictive of later receptive language skills (Choi et al. 2020). Lower rates of initiating joint attention are also observed in infant siblings with later autism (Mundy & Bullen 2021).

Neural sensitivity to eye gaze has been linked to activity of the temporal lobe (fusiform gyrus, STS, and amygdala) (Hooker et al. 2003). White matter pathways connecting the amygdala to the ventral-medial prefrontal cortex and anterior temporal pole in 6-month-old infants have been associated with later joint attention skills at 9 months (Elison et al. 2013b). Weaker functional connectivity between the cortex and cerebellum at 9 months of age predicted later language and joint attention skills and autism-related behaviors in infant siblings (Okada et al. 2022).

### Sensory and Motor Differences, Including Object Use

Differences in sensory processing, including hyper- and hyposensitivity to visual, auditory, and tactile stimuli, are observed at 6 months in infants with later autism (Sacrey et al. 2015, Wolff et al. 2019). Differences in how objects are used, such as intense visual inspection, repetitive play, and reduced exploration, are observed by 9–10 months in infants with later autism (Miller et al. 2021). These differences persist to 12 months of age, when stereotyped body and head movements are also observed (Dimian et al. 2017).

On the basis of MRI at 6 months, fractional anisotropy of the genu in the anterior corpus callosum and cerebellar pathways predicted later levels of repetitive behaviors and sensory responses, respectively, in infant siblings later diagnosed with autism (Wolff et al. 2017). Reduced neural repetition suppression to tactile input in 10-month-old infant siblings was associated with later autism (Piccardi et al. 2021).

Differences in motor development are present by 6 months and likely earlier (Dawson et al. 2000). By 6–9 months, infant siblings later diagnosed with autism exhibit delayed sitting, pull-to-sit, reach-to-grasp, and goal-directed reaching. By 12 months, fine and gross motor delays exist and predict later expressive and receptive language abilities (LeBarton & Landa 2019). Difficulties in postural control and frequent head movements appear early and continue into toddlerhood (Krishnappa Babu et al. 2022).

In 12-month-old neurotypical infants, fMRI functional connectivity of motor and default mode networks has been found to be associated with onset of walking, whereas dorsal attention and posterior cingulo-opercular networks are associated with gross motor skills by 24 months, underscoring the dynamic nature of brain changes and motor development (Marrus et al. 2018). Little is known about the brain systems related to motor development in infants with later autism. The correlation between early motor skills and later language acquisition underscores the involvement of cortical motor regions in the production of syllabic vocalizations (Bouchard et al. 2013).

### Summary

Research based on home videotapes, clinical case reports, and observations of infant siblings has demonstrated that, during the period from 6–12 months, infants later diagnosed with autism exhibit differences in four behavioral domains: (*a*) attentional flexibility, (*b*) social attention and engagement, (*c*) prelinguistic development, and (*d*) sensorimotor behavior. These domains represent focus areas for early intervention and are highly integrated such that development in one domain (e.g., attentional flexibility) impacts others (e.g., joint attention).

Studies examining the neural correlates of behavioral precursors of autism underscore the widespread differences in brain development apparent during infancy. fMRI studies have documented differences in the functional connectivity of cerebellum corticospinal pathways, splenium, corpus callosum, auditory and somatosensory regions, amygdala, anterior temporal cortex, and ventral-medial prefrontal cortex. EEG studies have pointed to differences in the neural networks supporting face, eye gaze, and phoneme processing and habituation to sensory stimuli involving the temporal lobe, amygdala, STS, and fusiform gyrus. The neural systems related to behavioral precursors of autism are highly interconnected and mutually influential during development.

## PHYSICAL HEALTH DURING INFANCY

Autism is associated with higher rates of co-occurring medical conditions (Alexeeff et al. 2017), and many of these conditions are present during the first year of life (Engelhard et al. 2020). Preterm delivery, low birth weight, and perinatal stroke due to hypoxia are associated with higher rates of autism (Carlsson et al. 2022). A retrospective study using electronic health records found that, before 12 months, infants later diagnosed with autism are more than three times as likely to visit an ophthalmologist, gastroenterologist, or neurologist than those without an autism diagnosis (Engelhard et al. 2020). Another study reported that, in the first 3 years after birth, infants and toddlers later diagnosed with autism had increased rates of neurological; nutrition-related; genetic; ear, nose, and throat; and sleep conditions (Alexeeff et al. 2017). Compared with neurotypical infants, 10-month-old infant siblings have more night awakenings and longer sleep-onset latencies (De Laet et al. 2022). By 6–12 months, infants diagnosed with autism have higher rates of caregiver-reported sleep-onset problems, which have been associated with differences in hippocampal volume trajectories (MacDuffie et al. 2020).

A holistic intervention approach that promotes behavioral development while simultaneously addressing existing medical conditions could enhance the efficacy of infant autism interventions. For example, evidence suggests that unique changes in sleep physiology and patterns occur during infancy, which influence structural and functional brain development (Lokhandwala & Spencer 2022). Sleep also enhances learning via sleep-dependent memory consolidation in young children (Spanò et al. 2018).

## FINDINGS FROM STUDIES EVALUATING THE EFFICACY OF INFANT AUTISM INTERVENTIONS

Research on autism intervention with infants ≤12 months is still in its early stages, with relatively few randomized clinical trials with replicated findings (see Supplemental Table 2 for more detail on published studies). The first intervention studies with infant siblings and/or infants exhibiting autism-related behaviors were case reports (Green et al. 2002, Vismara 2008) and small feasibility studies with sample sizes ranging from 3–17 (Baranek et al. 2015, Green et al. 2013, Koegel et al. 2014, Rogers et al. 2014, Steiner et al. 2013). These showed encouraging results with improvements found in language abilities and functional communication (Baranek et al. 2015, Koegel et al. 2014, Rogers et al. 2014, Steiner et al. 2013), social engagement (Koegel et al. 2014, Rogers et al. 2014), sensory responsiveness, and the quality of caregiver–infant interaction (Baranek et al. 2015).

Randomized clinical trials of caregiver-mediated interventions for infants ≤12 months range from small to adequately powered, with varying results. In a study of 1-year-olds exhibiting autism-related behaviors (*N* = 87), Watson et al. (2017) found minimal effects on child outcomes of a caregiver-coaching intervention, Adaptive Responsive Teaching (ART) (30 sessions across 8 months). However, caregivers showed greater responsiveness to their infant’s cues after the intervention, and changes in responsiveness mediated the positive effects of the intervention on children’s communication abilities and sensory processing. In a randomized trial with 9-month-old infant siblings evaluating a caregiver-coaching intervention [an adapted version of Video-Feedback Intervention to Promote Positive Parenting (VIPP); *N* = 54, 12 sessions across 5 months] (Green et al. 2015, Juffer et al. 2017), caregivers in the intervention group showed less directiveness during interactions with their infants. Infants who received the intervention showed increased attentiveness to their caregiver and exhibited fewer autism-related behaviors, faster attention disengagement on the gap-overlap task, and improved caregiver-reported social abilities. A follow-up assessment of the same sample at 3 years of age found the intervention was associated with fewer autism-related behaviors and increased attentiveness and communication initiations during caregiver–infant interactions (Green et al. 2017). Although the study was not adequately powered to detect group differences in autism diagnoses, a higher percentage of children were diagnosed with autism in the intervention group [4 of 27 participants (15%)] compared with the nonintervention group [2 of 26 participants (8%)]. This pattern of results is consistent with improving outcomes without changing rates of clinical diagnosis. No effects were found on standardized assessments of cognitive and language abilities.

In a larger (*N* = 103) randomized clinical trial of the same caregiver-coaching intervention (adapted VIPP, 10 sessions across 5 months) with 12-month-old infants exhibiting signs of autism, the earlier effects of intervention on levels of autism-related behaviors were not replicated, and there were no intervention effects on caregiver–infant interactive style, infant attentiveness, or standardized measures of language and cognitive ability (Whitehouse et al. 2019). However, a follow-up assessment of this sample at 3 years of age found that children in the intervention group had reduced odds of receiving an autism diagnosis [3 of 45 participants (6.7%)] compared with the usual care group [9 of 44 participants (20.5%)] (Whitehouse et al. 2021). Positive effects of caregiver responsiveness continued but attenuated over time, and there were no intervention effects on infant attentiveness, infant positive affect, or standardized assessments of language and cognitive abilities.

### Measuring and Testing Neural Correlates in Infant Intervention Studies

To our knowledge, only two intervention studies have examined whether an autism intervention with infants was associated with measurable changes in infant brain activity. A randomized trial of a caregiver-mediated intervention (VIPP) with 9-month-old infant siblings (Green et al. 2015) included an auditory oddball ERP designed to measure the infant’s ability to detect changes in speech sounds and the gap-overlap task to measure attentional flexibility. Results showed no significant intervention effects for the auditory ERPs, although there was a trend toward reduced neural responsiveness to speech sounds in the intervention group. A moderate positive effect was found on attention disengagement, indicating the intervention resulted in increased infant attention flexibility.

Another study collected measures of face encoding (habituation) and two EEG measures (ERPs to faces and toys and spontaneous EEG while watching dynamic social and nonsocial videos) at 6, 12, and 18 months from a group of infant siblings (*N* = 33) (Jones et al. 2017). Between 9 and 11 months, infants were randomized to receive a caregiver-mediated intervention, Promoting First Relationships, which has been shown to increase caregiver responsiveness and contingent responding (*N* = 19) (Booth-LaForce et al. 2020) versus ongoing monitoring without intervention (*N* = 14). Compared with infants who did not receive the intervention, infants in the intervention group exhibited faster habituation to faces, ERPs (P400) to faces that were more comparable to a neurotypical group of comparison infants, and increased frontal EEG theta power (a measure of attentional engagement) while watching dynamic videos. This study is encouraging in suggesting that a relatively brief caregiver-mediated intervention can promote neural systems involved in social processing.

### Summary

Current evidence reveals mixed results from preliminary trials and randomized clinical trials evaluating the efficacy of interventions for infants with a higher likelihood of an autism diagnosis, delivered with infants ≤12 months. This conclusion is broadly consistent with a meta-analysis of the efficacy of very early autism interventions on neurodevelopmental outcomes for infants and toddlers under 3 years, which suggested there is low-to-moderate certainty evidence that such interventions have limited impact on neurodevelopmental outcomes by age 3 (McGlade et al. 2023). Despite encouraging findings from smaller, preliminary studies, a larger randomized clinical trial of a caregiver-coaching intervention with 12-month-old infants showed no clear benefit immediately after the intervention. When children were followed up with at 3 years of age, children who received the intervention exhibited fewer autism-related behaviors and were less likely to receive an autism diagnosis, but no positive benefits were found in terms of children’s cognitive, language, or adaptive behavior, domains that have been correlated with positive long-term outcomes. Evidence to date suggests that more research is needed to refine and further test the efficacy of infant autism interventions before recommendations for their broader use in clinical practice can be made. Few studies to date have included brain-based predictor and/or outcome measures. Inclusion of brain-based moderators, mediators, and outcomes will provide a better understanding of the neural systems involved in infant interventions.

## A DEVELOPMENTAL SOCIAL NEUROSCIENCE PERSPECTIVE ON INFANT AUTISM INTERVENTION

### Therapeutic Strategies Used in Infant Autism Interventions

Progress in developing effective infant autism interventions will require a better understanding of the essential components of such interventions and how they relate to early infant brain development. Therapeutic approaches commonly used in infant autism interventions draw heavily on developmental science, particularly the notion that joint engagement in caregiver–infant interactions is fundamental for early social and communication development. Distinct from joint attention, joint engagement occurs when the infant and caregiver are actively focusing on shared activities and usually involves affective sharing (Adamson et al. 2004). Joint engagement is essential for promoting the development of social brain networks (Dawson et al. 2005, Johnson et al. 2005).

Most infant autism interventions use caregiver coaching, often delivered in weekly sessions for several months, with a core focus on increasing caregiver sensitivity and responsiveness to infant cues to support joint engagement. In neurotypical development, caregiver responsiveness has been found to enhance joint engagement and the acquisition of joint attention skills and language (Tomasello & Farrar 1986). Studies of young autistic children have shown that the frequency of caregivers’ contingent responses to the child’s actions and utterances is associated with rate of language acquisition (McDuffie & Yoder 2010). Importantly, during caregiver–infant interactions, the infant’s responses influence caregiver responsiveness, which in turn can have cascading effects on social and language development, known as the social feedback loop (Warlaumont et al. 2014).

Infant autism interventions encourage caregivers to adapt their interaction style to enhance the fit between the infant and their unique way of processing information and their environment, with a goal of optimizing the infant’s joint engagement and learning. For example, VIPP (Juffer et al. 2017, Whitehouse et al. 2021) uses video feedback to encourage the caregiver to recognize the pace and pattern of the infant’s behaviors and match their own responses, thereby increasing synchrony. Reciprocal vocalizations are supported by promoting contingent, attuned responses to the infant. In Infant Start (Rogers et al. 2014), based on the Early Start Denver Model (Rogers et al. 2019), caregivers are coached to position themselves for optimal face-to-face orientation, follow the infant’s interests and preferred activities, respond contingently by imitating and elaborating on the infant’s behavior, optimize the infant’s state of arousal and positive affect, and maximize opportunities for positive shared affect and turn-taking. In the ART model (Watson et al. 2017), caregivers are coached to use responsive strategies involving contingency, matching, and affective responding. Infant interventions based on Pivotal Response Training encourage caregivers to pair themselves with the infant’s preferred activity to increase the infant’s motivation to engage in social interaction, interspersing highly preferred activities that are associated with positive affect with more neutral activities (Koegel et al. 2014).

In these examples, caregivers and the coach reflect together on the interaction between the infant and caregiver and discuss strategies designed to enhance social engagement and communication. Common strategies include (*a*) increasing the salience of social information by removing distracting objects and positioning themselves to maximize face-to-face interaction, (*b*) following the infant’s lead by engaging in infant-preferred activities rather than directing the interaction, thereby following the pace and interests of the infant, (*c*) responding contingently to the infant’s behaviors, often by mirroring the infant’s facial expressions, vocalizations, and actions, and (*d*) increasing enjoyment of the shared experience by participating in infant-initiated activities and being sensitive to the infant’s affective state and arousal levels. These elements are among those identified as common to naturalistic developmental behavioral interventions that have been shown to be effective with toddler- and preschool-aged autistic children (Frost et al. 2020).

### Implications of Developmental Social Neuroscience Theories of Autism for Understanding Infant Autism Interventions

We now consider how contemporary developmental social neuroscience theories of autism can shed light on the therapeutic strategies used in infant autism interventions. These theoretical perspectives on brain function in autism are not mutually exclusive but rather are expected to interact in synergistic ways.

#### Predictive coding hypothesis.

The predictive coding hypothesis posits that autism affects the infant’s ability to make predictions about sensory inputs and evaluate the correspondence between predictions and experience in order to build a coherent model of the social world (Cannon et al. 2021). The acquisition of social and communication abilities depends on the ability to perceive predictable patterns in the environment via pattern learning (Faust et al. 2020). Infants detect and learn patterns of sensory information, including speech, actions, and visuospatial patterns, by noticing recurrent regularities (e.g., co-occurrence probabilities and distributional properties), which are then generalized to other situations (Saffran 2020). Through experience, infants track the patterns present in their environment to identify meaningful distinctions in the sensory input around them that allow them to process speech and faces (Krasotkina et al. 2018).

Contingency detection is a fundamental way in which infants use statistical learning to develop social and communicative skills, including the ability to predict another person’s actions, which is central to joint attention. Neurotypical infants whose caregivers provide contingent vocal responses to their babbling are more likely to incorporate their caregiver’s phonological patterns compared with infants who receive the same number of noncontingent responses (Goldstein & Schwade 2008). Other contingent responses to babbling, such as smiling or being touched, also increase the number and quality of infant vocalizations. As infants notice predictable actions of their caregivers, this influences their attentional focus and preferences. Infants prefer and attend more to actions by their caregiver that are reliable (Tummeltshammer & Kirkham 2013). Infants who receive a high ratio of jointly focused contingent responses by caregivers prefer the objects their caregiver is holding, thus facilitating shared attention (Mason et al. 2019b). For these reasons, contingent responding is a key intervention strategy that supports the development of infant self-regulated attention. However, social responding by others is rarely perfectly contingent. Thus, promoting a tolerance, or even a preference, for high but imperfect contingency is also important.

Detecting and learning statistical regularities in the context of social interaction occurs via predictive processing. Contemporary computational neuroscience models posit that a fundamental principle of brain function and learning is to minimize prediction errors (Clark 2013). Predictions are made within a hierarchical system from motor responses to mental representations and include predictions about one’s own actions and the actions of others, as well as shared actions, rewards, and emotions (Brown & Brüne 2012). Mismatches between prediction and outcomes are opportunities for learning. Predictions involving biological motion that include inferences about the goals of others’ actions involve the STS ( Jellema et al. 2000) and mirroring network (Molenberghs et al. 2012). The mirroring network, which plays a central role in forming shared action representations, is a widely distributed brain system involving the inferior parietal lobe, inferior frontal gyrus, ventral premotor cortex, primary visual cortex, cerebellum, and parts of the limbic system (Molenberghs et al. 2012). Several of these brain regions have been implicated in studies of infants with a later diagnosis of autism (Elison et al. 2013b, Okada et al. 2022, Wolff et al. 2017).

Bayesian accounts of autism hypothesize that differences in predictive processing underlie autism-related behaviors (Sinha et al. 2014, Van de Cruys et al. 2014). Evidence supporting this model of autism is summarized by Palmer et al. (2017) and Cannon et al. (2021). From a Bayesian perspective, social interaction is challenging for autistic persons because of the high degree of uncertainty (unpredictability) of human behavior, which inherently involves a greater number of prediction errors, especially if predictions (Bayesian priors) are imprecisely matched to sensory inputs (Lawson et al. 2014). This perspective posits that any stimulus or environment involving a high degree of uncertainty will be challenging, not solely those of a social nature.

The predictive coding hypothesis posits that, whereas neurotypical infants apply differential weights to prediction errors during social interactions to determine whether new learning is necessary, the infant who will be diagnosed with autism assigns disproportionate weight to nonrelevant prediction errors, resulting in a higher number of instances in which the infant perceives the social input as being novel. According to this view, the neurotypical infant uses aggregated input to assign weight to prediction errors, while the infant with early signs of autism places higher weight on the most recent input, such as immediate sensory information (Köster et al. 2020). This not only would affect how the infant makes sense of social and linguistic information but also could influence sensory processing (van Laarhoven et al. 2020) and help explain findings that infants with later autism fail to suppress neural responses to repeated sensory stimuli (Piccardi et al. 2021). Studies of 6-month-old neurotypical infants show that infants’ neural responses to repeated auditory stimuli are modulated by the degree of predictability when they experience such stimuli (Emberson et al. 2019). A tendency to place higher weight on recent sensory input could also lead to overselectivity, involving local versus global processing biases (Leader et al. 2009).

Early differences in predictive processing would also be expected to influence the development of multisensory representations of peripersonal space and shared action spaces (Noel et al. 2022, Pezzulo et al. 2013). Infants continuously form models to predict both their own and another’s actions, successively integrating multisensory information to form an action plan that includes both partners, which is necessary for real-time coordination. Autistic individuals have been found to have diminished ability to update representations of their peripersonal space in response to a changing social context (Noel et al. 2022).

#### Multisensory temporal perception hypothesis.

Recent conceptualizations of autism have proposed that altered multisensory processing may help explain many core features of autism, including social and communication difficulties and sensory sensitivities (Siemann et al. 2020). Multisensory processing involves integrating sensory input from different modalities to form a unified perception, which can enhance perception, especially in noisy environments (Stevenson et al. 2014). Numerous studies have documented differences in multisensory processing in autistic individuals (Siemann et al. 2020), including decreased strengthening of speech perception when congruent auditory and visual information is provided (i.e., the McGurk effect) (Irwin et al. 2011). Research suggests that the multisensory temporal binding window might be unusually wide in autistic individuals (Foss-Feig et al. 2010). Alterations in temporal processing of multiple stimuli might change how the brain differentially weights two inputs and whether they should form an integrated percept. In support of this perspective, reduced visual orienting to audiovisual synchrony in 10-month-old siblings predicted a later autism diagnosis (Falck-Ytter et al. 2018).

Integration of multimodal information can enhance speech perception and has a central role in language learning (Mason et al. 2019a). Multisensory processing of speech has been found to involve the STS, a highly interconnected hub brain region that has also been shown to have both structural and functional differences in neuroimaging studies of autistic individuals (Redcay 2008). As a multimodal and rapid temporal integration hub, the STS may be differentially engaged by social stimuli as their complexity necessarily requires its computational ability, although the debate about the degree to which it is dedicated only to social stimuli continues (Lahnakoski et al. 2012). Infant-directed speech by the caregiver, characterized by auditory prosody exaggerations, is often temporally synchronized with exaggerated visual facial movements and expressions (Green et al. 2010). Similarly, caregivers’ use of gestures and object movements is often temporally synchronized with infant-directed speech and word labels. Studies of neurotypical infants highlight the importance of the caregiver’s role in coordinating their speech with the infant’s attention and actions. During the first year after birth, infants are much better at extracting information from their caregivers, including word learning, when the caregiver responds to the infant’s prelinguistic vocalizations within a 2–5 second time window (Goldstein & Schwade 2008).

Multisensory integration has also been shown to modulate arousal. In a study of 4-month-old neurotypical infants, electrodermal activity was reduced when the infant experienced a combination of a socially relevant visual stimulus and affective touch (Nava et al. 2021). Thus, for infants with early signs of autism, difficulties in multisensory temporal integration would be expected to impact sensory processing, arousal levels, perception of facial expressions and speech, and early vocalizations and language learning.

#### Social motivation hypothesis.

Social motivation accounts of autism posit that autism involves differences in evolutionary biological mechanisms that bias an infant to preferentially orient to social information (social orienting) and to approach and experience reward from social interaction (Chevallier et al. 2012, Dawson et al. 2002, Dawson et al. 2005, Schultz et al. 2000). Neural circuitry involved in social motivation includes the orbitofrontal-striatal-amygdala circuit and is mediated by neuropeptides such as oxytocin and vasopressin, as well as other neuromodulatory systems involved in reward, such as dopamine and the endocannabinoid system (Walum & Young 2018). Recent evidence suggests that differences in reward salience and activation in autism are not specific to social stimuli (Keifer et al. 2021). A meta-analysis of fMRI studies during processing of social versus nonsocial rewards by autistic individuals supports a broader interpretation of the social motivation theory that implicates both social and nonsocial reward (Clements et al. 2018). From this point of view, the infant with early signs of autism would be expected to have reduced motivation to engage in both social and nonsocial aspects of their environment.

The role of social motivation during infancy in autism is supported by reduced attention to faces, positive affect, and eye contact during social interaction (Bradshaw et al. 2021, Garon et al. 2022, Macari et al. 2021) and diminished response to name (Hatch et al. 2021, Miller et al. 2017) in infants later diagnosed with autism. Cortical networks supporting social attention develop during infancy, including the salience network (anterior insula and dorsal anterior/midcingulate cortex), which allocates attention to preferred stimuli, along with its connection to the amygdala, which plays a key role in emotion, arousal, and motivation (Gao et al. 2015). In autism, suboptimal states of arousal (hyper- or hypoarousal) could influence attention to and encoding of social information and acquisition of social and communication skills (Tonnsen et al. 2018).

A study of resting-state functional connectivity of neurotypical infants found that differences in connectivity between the amygdala and salience network mediated the relationship between caregiver affect and infant smiling (Phillips et al. 2021), supporting the role of these networks in affective and social engagement during infancy. Differences in patterns of early amygdala growth have been found in infant siblings with a later diagnosis of autism (Shen et al. 2022).

### Common Elements of Infant Autism Interventions Reconsidered

The predictive coding hypothesis suggests that the infant with a later diagnosis of autism exhibits reduced social engagement and positive affect because of the high degree of uncertainty in social behaviors, leading to a high level of prediction errors, which are experienced as novel events. The infant might overly weight information not directly relevant to the social interaction, making it more difficult to detect statistical regularities in social patterns, such as the caregiver’s actions, speech, and facial expressions. A common intervention strategy is to minimize attention to nonessential information and emphasize the information that is most relevant by removing distracting information and increasing the salience of the key information most relevant to social interaction (e.g., encouraging face-to-face positioning where the caregiver is in the center of the infant’s visual field).

By responding in a highly contingent manner to the infant’s behavior, including mirroring the infant’s behaviors, the caregiver provides a highly predictable interaction and reduces noise and uncertainty. The use of songs and games, often incorporated into caregiver-mediated interventions, provides highly predictable, repeatable social experiences. As the infant notices the statistical regularity of the contingent responses, the infant will often begin testing these predictions by intentionally vocalizing or moving to see if this elicits a response from the caregiver. In this way, the infant’s increased use of social behaviors facilitates responses from the caregiver, thereby enhancing the social feedback loop. Another strategy is to establish highly predictable joint activity routines and then slowly introduce variation into the joint activity while still maintaining a relatively high level of predictability. These types of experience are expected to stimulate a wide range of neural networks (e.g., salience and mirroring networks, networks supporting face processing). In an intervention study of toddlers diagnosed with autism, children who received a naturalistic developmental behavioral intervention (Early Start Denver Model) exhibited a faster ERP response and increased cortical activation (reduced alpha and increased theta spectral power) when viewing faces (Dawson et al. 2012) and increased activity of the mirroring network, as evidenced by increased EEG mu rhythm suppression while watching an action performed by a familiar person (Aaronson et al. 2022).

The multisensory temporal perception hypothesis posits that the infant showing early signs of autism has difficulty processing social information due to its multisensory and temporal nature. The infant is not readily integrating the caregiver’s facial expressions and actions with their words and sounds, thus diminishing perception of this social information. If the infant has an unusually wide time window for multisensory temporal binding, the infant might mistakenly integrate information that is not meaningfully related (e.g., facial expression with a background sound). Increasing the salience and the regular temporal concurrence of relevant visual (the caregiver’s facial expressions) and auditory (vocalizing to the infant) information would potentially increase the chances of temporal binding. Simultaneous mirroring of the infant’s body movements might promote the infant’s ability to integrate proprioceptive information with the visual information of watching the same movement in another person. Songs and infant games provide opportunities for experiencing high temporal congruence of movement, sound, and visual input.

The social motivation hypothesis suggests that diminished social attention and engagement are influenced by a reduced level of reward assigned to social experiences. This perspective puts high value on the infant experiencing the person and interaction as pleasurable. Following the infant’s lead and participating in the infant’s self-initiated and preferred activities ensures that the infant already has a high level of interest in the shared activity. Associating preferred activities, such as songs, physical games, and toys, with the caregiver (paired-associate learning) enhances the reward value of the caregiver. Sensitivity to the infant’s affective state and arousal level maximizes opportunities for attentional engagement and shared positive affect and minimizes experiences that are overly arousing and stimulating for the infant. From this point of view, increasing the reward value of social interaction is expected to naturally draw the infant’s attention to the caregiver’s face, voice, and actions, thereby stimulating activity in brain networks involved in face and voice processing (Dawson et al. 2002).

**Figure 1:**
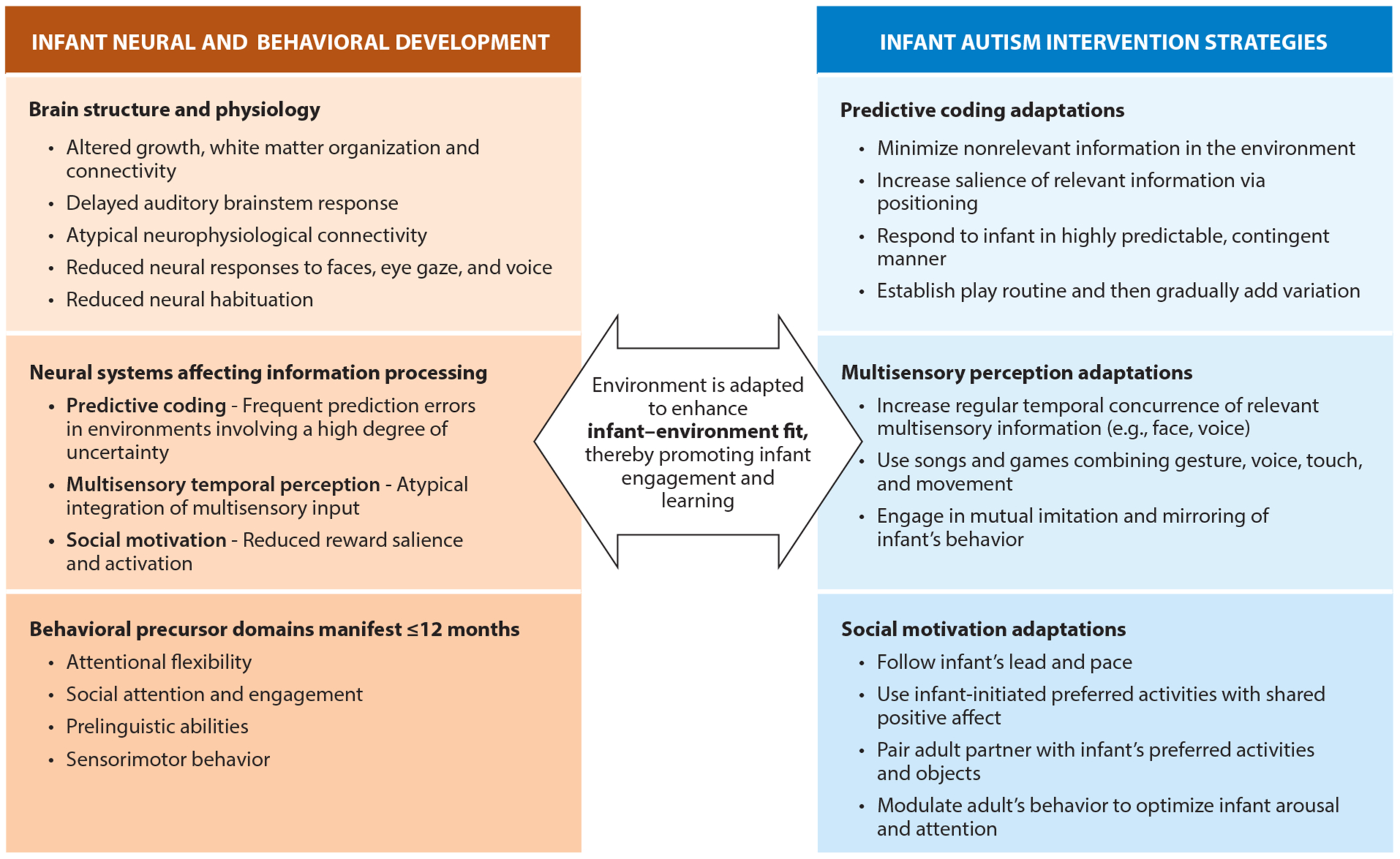
Enhancing infant–environment fit to promote social engagement and learning. A wide range of genetic and physical health factors beginning during the prenatal period lead to changes in brain development, including brain growth, specialization, connectivity, and responsiveness to various stimuli. These are manifest in infant information processing differences, including differences in predictive coding, multisensory temporal perception, and social motivation, and multiple behavioral precursors to autism, observable by 6–12 months of age. Infant autism interventions involve therapeutic adaptations to the infant’s unique style of information processing, with a goal of enhancing infant–environment fit, thereby promoting infant social engagement and learning.

In summary, contemporary developmental social neuroscience provides a strong foundation for current infant autism interventions, illustrated in Figure 1. We posit that the goal of infant autism intervention is for the caregiver to learn to make adaptations to the infant’s social and nonsocial environment to enhance infant–environment fit (Edwards et al. 2006). A higher degree of fit is expected to promote social engagement and learning. In this sense, both the infant and their caregiver are mutually engaged in a dynamic process of adaptation to optimize development and outcomes for the infant.

## FUTURE DIRECTIONS AND CONCLUSIONS

### Considerations for Designing Future Intervention Studies

We review above the state of the evidence supporting the efficacy of autism infant intervention for improving children’s outcomes and describe current approaches to infant autism interventions from the perspective of contemporary neuroscience theories of autism. We now consider how a developmental social neuroscience perspective can inform future directions for infant autism intervention studies and discuss ethical considerations raised by infant autism interventions.

#### Using brain-based measures to inform individualized approaches to intervention.

Given that autism is a heterogeneous condition, tailoring interventions to the specific needs of each individual may improve outcomes, as individual infants may benefit from adaptations of interventions to their unique way of interacting with others and the broader environment (Hartman & Belsky 2016). Incorporating infant neural biomarkers or behavioral precursor profiles may identify infants who will most benefit from specific types or intensities of intervention. Table 1 describes several neural correlates of behavioral precursors of later autism that are developmentally appropriate for use in infant intervention studies. Incorporation of such neural correlates into infant intervention clinical trials could elucidate which interventions work for which infants and enhance our understanding of the neural mechanisms underlying behavioral change in response to intervention.

#### Better established biomarkers.

To better understand the effects of interventions and their influence on underlying brain systems, we need robust biomarkers of these systems. We discuss above a variety of behavioral, electrophysiological, and neuroimaging measures that have had utility in predicting a later diagnosis of autism. Some of these measures are conceptually linked with infant behavioral precursors to autism and would be expected to change along with behavioral improvements (see Table 1). Currently, there are no formally established biomarkers for autism. However, one electrophysiological measure has been the subject of preliminary qualification as a stratification biomarker by the European Medicines Agency and the US Food and Drug Administration: the N170 ERP response to faces (Kala et al. 2021, Mason et al. 2022, McPartland et al. 2020). As discussed earlier, several infant studies have implicated neurophysiological responses to faces and other social stimuli as associating with later autism, at least as a group predictor (Jones et al. 2016, Lloyd-Fox et al. 2018), and prediction is strengthened when combined with genetic factors (Gui et al. 2021b). Further exploration of this infant brain marker for social attention and engagement is merited and promises to index activity consistent with multisensory temporal perception (STS being one component of the core face network) and/or social motivation systems. Similarly, reduced social attention measured via eye tracking shows promise as a viable autism biomarker (Shic et al. 2022). Recent studies based on computer vision analysis have demonstrated the ability to detect differences in social attention in autistic toddlers on the basis of eye-gaze patterns using an application administered on a smartphone or tablet in clinics or homes (Chang et al. 2021). These novel computational approaches offer promise for scalable, quantitative social attention biomarkers that can be collected remotely in natural settings.

#### Time course of intervention.

To date, interventions have typically focused on the age range of 7–12 months and usually involved 10–12 sessions over 3–5 months. A strong possibility is that a more prolonged period of intervention, or a tiered approach in which intervention continues if little or no change occurs, would yield stronger intervention effects. Given our understanding of infant brain development in autism and the potential impact of early physical health conditions, another option for future studies is to begin intervention at an earlier stage, even during the neonatal period. Recruiting infants to intervention only after screening reveals early emerging autism signs may result in a missed optimal window for commencing intervention. Future research is needed to better understand when the optimal time is to begin intervention and how to monitor progress and adapt interventions to address the individual needs of each infant and their family. An intervention study with autistic toddlers suggests that even a small difference in the timing of intervention onset (18 versus 27 months) may influence developmental outcomes (Guthrie et al. 2023).

#### Multifaceted intervention, including infant-directed intervention.

Current interventions have focused on caregiver–infant interaction as the route to effect change. However, based on examples from other conditions, it is plausible that a multifaceted intervention that includes infant-directed components would be more successful. A broader array of interventions can address multiple developmental pathways that may have interactive or additive influence on the infant’s trajectory. For example, disrupted sleep patterns and other physical health difficulties are known to have detrimental effects on learning and affect and thus could cause or compound social interaction difficulties. Other infant-directed interventions using new technology could also facilitate the development of underlying brain systems. Trials of attention training using gaze-contingent eye tracking have been conducted with neurotypical 11-month-old infants (Wass et al. 2011). The infants interacted with a series of enjoyable games in which their looking patterns determined the presentation of attractive rewarding stimuli in a contingent way. Relative to a passive-viewing control group, the intervention group showed positive change in cognitive control and sustained attention, saccadic reaction times, and attention flexibility. However, a subsequent proof-of-concept randomized controlled trial conducted in more naturalistic settings failed to replicate these findings (Goodwin et al. 2021). Results from ongoing trials and longer-term follow-ups are yet to be reported. If proven effective, such infant-directed interventions could be conducted alongside a caregiver-mediated intervention.

### Ethical Considerations

Early neurodevelopmental studies and intervention trials require particular ethical considerations. Infant autism research gives rise to questions about the potentially conflicting rights and priorities of the infants concerned, caregivers and families, and autistic adults (Manzini et al. 2021). While for scientists, research into early detection and predictors in early infancy is sometimes motivated by a desire to understand early causal pathways to later autism, for the participating families its value lies in the opportunity for subsequent intervention to improve outcomes for their child. As discussed earlier, progress is being made to develop stronger individual infant predictors of later autism, and this will consequently reduce the likelihood of false alarms and missed cases. The risk associated with false positives (intervening when unnecessary) can also be mitigated through the use of more generic interventions, which are thought to be beneficial for a broad range of infants and have few, if any, known adverse effects (van Leeuwen et al. 2020). It is recommended that studies of infant autism intervention monitor and report adverse effects to better assess both the benefits and potential harms of such interventions.

Historically, autism has been viewed from a biomedical model, with interventions prior to diagnosis intended to reduce the likelihood of a later diagnosis (Whitehouse et al. 2021). In contrast, proponents of the neurodiversity movement argue that defining autism in terms of impairments and deficits, without consideration of its positive traits, is discriminatory. From this point of view, the purpose of intervention should be to adapt the infant’s environment to better fit the infant’s unique profile with the goals of promoting social, language and communication, self-regulation, and adaptive skills and nurturing autistic strengths (Dawson et al. 2022).

Future consideration should be given to the selection of primary outcomes for intervention trials. We recommend a focus away from later autism diagnosis as a target for change and a shift toward measures that reflect the child’s communication and learning skills, self-regulatory and adaptive behavior, and quality of life (Dawson et al. 2022). Ideally, the intervention should be associated with meaningful and sustained change in distal measures that indicate generalization of abilities across environments (unbounded) rather than solely a change in the skills directly targeted in the intervention (proximal) (Franz et al. 2022). Following infants into childhood and assessing levels of anxiety and other mental health conditions will also be important, as these outcomes significantly impact quality of life. Finally, children from some infant studies are now old enough to be interviewed. Their views and those of others with lived experience should be considered in planning future research studies (Manzini et al. 2021).

## Supplementary Material

Supplimental table 1

Supplemental table 2

## Figures and Tables

**Table 1:** Potential infant brain-based and attention measures for use in clinical trials evaluating infant autism interventions

Behavioral precursor	Earliest age documented in infants later diagnosed with autism (months)	Potential infant brain-based biomarker
Reduced attention to faces and gaze following (Macari et al. 2021)	6	ERPs to faces ( Jones et al. 2016) Intertrial coherence during face processing (van Noordt et al. 2022) Social attention–related EEG microstates (Gui et al. 2021a) Eye tracking of social gaze (Chang et al. 2021, Jones & Klin 2013)
Lower positive affect (Garon et al. 2022)	6	EEG alpha asymmetry during reward anticipation (Stavropoulos & Carver 2014) Frontal EEG asymmetry (Campagna et al. 2021)
Multisensory and sensory differences (Sacrey et al. 2015)	6	Orienting to multisensory stimuli (Falck-Ytter et al. 2018) Repetition suppression of evoked gamma (Kolesnik et al. 2019) Somatosensory mismatch negativity (Shen et al. 2018) Pupillary light reflex (Nyström et al. 2018)
Motor imitation (LeBarton & Landa 2019)	6	EEG mu rhythm suppression to familiar action (Marshall & Meltzoff 2011)
Increased negative affect (Pijl et al. 2019)	8	Frontal EEG asymmetry (Campagna et al. 2021) Mu rhythm suppression to emotional faces (Quadrelli et al.2021) ERPs to emotional faces (Quadrelli et al. 2019)
Reduced gaze following (Bussu et al. 2021)	8	Predictive learning of goal-anticipatory gaze shifts (Gumbsch et al. 2021)
Reduced response to name (Hatch et al. 2021)	9	Mismatch negativity to speech sounds (Paquette et al. 2013) NIRS responses to human sounds (Braukmann et al. 2018)
Increased repetitive behaviors (Miller et al. 2021)	9	EEG functional connectivity (Haartsen et al. 2019)
Reduced social affective engagement (Bradshaw et al. 2021)	9	EEG mu suppression to emotional faces (Quadrelli et al. 2021) ERPs to emotional prosody (Grossmann et al. 2005) Frontal EEG asymmetry (Campagna et al. 2021)
Reduced canonical vocalizations (Yankowitz et al. 2022)	12	Mismatch negativity to speech sounds (Cheour et al. 2001) NIRS responses to human sounds (Braukmann et al. 2018)
Attention disengagement during play (Sacrey et al. 2013)	12	Gap-overlap task (Elsabbagh et al. 2009)
Reduced initiating joint attention (Franchini et al. 2019)	12	ERPs to eye gaze shifts (Bussu et al. 2021)

Abbreviations: EEG, electroencephalography; ERPs, event-related potentials; NIRS, near-infrared spectroscopy.

## SUMMARY POINTS

1. A wide range of genetic and pre- and postnatal physical health factors influence brain development in infants later diagnosed with autism.
2. Alterations in brain development in autism affect neural systems underlying information processing, including differences in predictive coding, multisensory temporal perception, and social motivation.
3. Changes in the way the infant processes information are manifest in behavioral precursors of autism, observable from 6–12 months of age.
4. Behavioral precursors to autism are observed in the domains of attention flexibility, social attention and engagement, prelinguistic behavior, and sensory and motor function.
5. The therapeutic strategies used by caregivers in most infant autism interventions, such as increasing the salience and predictability of social information, reflect specific environmental adaptations to the infant’s unique way of processing information designed to enhance the fit between the infant and their environment.
6. Clinical trials evaluating the efficacy of infant autism interventions have generated some positive findings, albeit with mixed results and a lack of replication.
7. Infant autism interventions can be consistent with a neurodiversity perspective on autism when a strengths-based approach is used and outcome measures focus on well-being and quality of life of the child and family.

## FUTURE ISSUES

1. Tailoring interventions to the specific needs of each infant could improve outcomes, as individual infants may benefit from distinct therapeutic strategies adapted to their unique way of interacting with others and the broader environment.
2. Incorporation of brain-based biomarkers into infant intervention clinical trials could elucidate which interventions work for which infants and enhance our understanding of the neural mechanisms underlying behavioral change in response to intervention.
3. Future studies of infant autism intervention should evaluate the effects of timing and dose of intervention and consider a tiered approach that adjusts these factors depending on the infant’s response to the intervention.
4. Multifaceted interventions that include infant-directed approaches, such as those using gaze-contingency eye tracking, might improve children’s outcomes if used alongside a caregiver-mediated approach.
5. Addressing the physical health conditions associated with autism present during infancy, such as sleep disruptions, could improve responses to caregiver-mediated interventions and children’s outcomes.
6. Future trials of infant autism interventions should include a broad range of outcomes, including assessments of social communication, language, learning, adaptive skills, autistic strengths, and overall quality of life.
7. Implementing autism interventions with infants raises important ethical considerations about the rights and priorities of the infants, caregivers and families, and individuals with lived experience, and as such, involving these stakeholders in the future design of infant intervention studies is important.
