# Supplementary material for: A Developmental Social Neuroscience Perspective on Infant Autism Interventions
Source: Annu Rev Dev Psychol. Author manuscript; Available in PMC 2025 Jun 13. (PMC12165438; doi:10.1146/annurev-devpsych-120621-042753)
Supplement: Supplimental table 1 [file NIHMS2084123-supplement-Supplimental_table_1.pdf]

A developmental social neuroscience perspective on infant autism intervention
Annual Review of Developmental Psychology
Geraldine Dawson, PhD, Amber D. Rieder, PhD, and Mark H. Johnson, PhD

Supplemental Table 1. Glossary of terms

Amygdala	An almond shaped collection of nuclei located within the temporal lobe that is involved in emotions, memory, and social behavior.
Anterior temporal pole	A region of the temporal lobe involved in semantic processing, social cognition, and emotional processing.
Auditory oddball event-related potential	A measure of brain activity in response to an infrequent, unexpected sound within a sequence of more frequent, expected sounds, that reflects the brain's processing of attention and cognitive control. Also see mismatch negativity.
Bayesian	Statistical method that assigns probabilities to events based on experience or estimates before data collection and applies Bayes' theorem to revise the probabilities after obtaining data.
Biological motion	Refers to the motion and movement patterns derived from the actions of an animal and its limbs, which are recognizable and distinguishable from motion of non-living objects.
Cerebellum	A part of the brain between the cerebrum and brainstem that controls balance, coordination, and movement.
Cingulate cortex	A part of the brain in the medial region of the cerebral cortex that is involved in emotions, learning, and decision-making.
Corpus callosum	A bundle of nerve fibers, or white matter tracts, that connect the left and right hemispheres of the brain. Also see genu of the corpus callosum.
Default mode network (DMN)	A network of brain regions active when the mind is at rest and not focused on external stimuli.
Dopamine	A type of neurotransmitter that has a key role in reward, motivation, movement, and other functions.
Endocannabinoid system	A network of chemical signals and receptors that controls activity of other neurotransmitters. This system is stimulated by molecules produced by our bodies (endocannabinoids) that stimulate receptors involved in immune function, as well as learning and memory.
EEG spectral power densities	Spectral EEG power analysis is used to quantify the amount of brain activity present during resting-state or presentation of various stimuli. It quantifies power in each frequency band (delta, theta, alpha, beta, and gamma) and can be represented as absolute or relative power (absolute power in each frequency band as a percentage of the sum of all frequencies).
EEG microstates	EEG microstates represent a dynamic assessment of the spatial and temporal distribution of the electrical signal across the scalp electrodes, defining a brief, quasi-stable state in any frequency band.

Supplemental Material: Annu. Rev. Dev. Psychol. 2023. 5:89-113
https://doi.org/10.1146/annurev-devpsych-120621-042753

A developmental social neuroscience perspective on infant autism intervention
Annual Review of Developmental Psychology
Geraldine Dawson, PhD, Amber D. Rieder, PhD, and Mark H. Johnson, PhD

EEG μ rhythm suppression	μ rhythm (8-13 Hz) suppression occurs when a person performs or views another person performing a motor action and is believed to reflect the activity of the mirror neuron system.
Event-related brain potential (ERP)	ERPs reflect the electrophysiological response to a specific stimulus generated by large groups of neurons, which is repeated and then averaged to reveal components time-locked to the stimulus presentation that vary in latency and amplitude.
Fractional anisotropy	Fractional anisotropy is used in diffusion tensor imaging research to reflect the integrity of white matter (degree of myelination and axonal density) ranging from 0 (weak) to 1 (strong).
Frontoparietal orienting network	A network mediated through the frontal and parietal lobe that is important for flexible allocation of attention and cognitive control.
Functional magnetic resonance imaging (fMRI)	fMRI measures changes in the blood oxygen levels in specific brain regions during resting state or stimulus or response time-locked (Blood Oxygenation Level Dependent; BOLD) to assess regional brain activity.
Fusiform face area (FFA)	A region in the inferior temporal lobe that is selectively engaged in face processing.
Gap-overlap task	A task that measures disengagement and shifting of visual attention between a central and peripheral stimulus and involving trials where the two stimuli do or do not overlap in time with each other.
Genu of the corpus callosum	Anterior part of the corpus callosum that connects the medial and lateral surfaces of the frontal lobes.
Hippocampus	A part of the brain located in the temporal lobe that plays a critical role in learning and memory.
Inferior frontal gyrus	A part of the brain that contains Broca's area which is involved in language processing and speech production.
Insula	A brain region separating the temporal lobe and inferior parietal cortex which is involved in sensorimotor, auditory, and vestibular functioning, among other functions.
Inter-trial EEG coherence	A measure of consistency of the phase angle of oscillatory activity measured using EEG across trials.
Mirroring network	Also called the "mirror neuron network," this is a group of specialized neurons that respond to the actions observed in other people.
Mismatch negativity	A component of the event-related potential responding to an odd, infrequent stimulus in a sequence of repeated stimuli.
Medial prefrontal cortex	A region of the prefrontal cortex located in the middle of the brain's frontal lobe, involved in a variety of complex cognitive and emotional processes, including decision-making, social cognition, and self-reflection.
Near infrared spectroscopy (NIRS)	Functional NIRS is a non-invasive technique that uses near-infrared light sources and detectors to measure hemodynamic responses (e.g.,

	oxygenation) in the brain, reflecting increases and decreases in regional neural activity.
Orbitofrontal cortex	A region in the prefrontal cortex in the frontal lobe of the brain involved in decision-making, emotions, and responding to reward.
Oxytocin	A peptide hormone produced by the hypothalamus that stimulates muscle contraction of the uterus during childbirth and lactation and is associated with affiliation, empathy, and trust.
Parietal lobe	A part of the cerebral cortex that processes somatosensory information and spatial awareness.
Proprioception	Related to stimuli that are produced and sensed within the body, such as the position and movement of the body.
Pupillary light reflex	Autonomic reflex that involves constriction of the pupil in response to sudden changes in light.
Saccade	Rapid movement of the eye between two fixation points.
Salience Network	A network in the brain involving the anterior insula and dorsal anterior/mid cingulate cortex that selects which stimuli should be prioritized for attention.
Splenium	A posterior section of the corpus callosum that connects the left and right cerebral hemispheres and facilitates communication between them.
Statistical learning	Learning by detecting the statistical structure (conditional probabilities) of events and elements in the environment.
Superior temporal sulcus (STS)	A region around the main groove in the temporal lobe, running parallel to the Sylvian fissure, that is involved in visual and auditory social perception such as processing gaze direction and emotional expressions.
Vasopressin	A hormone made by the hypothalamus involved in modulating the body's osmotic balance, blood pressure, and kidney function, as well as social affiliation and aggression.
