# Supplementary material for: A Developmental Social Neuroscience Perspective on Infant Autism Interventions
Source: Annu Rev Dev Psychol. Author manuscript; Available in PMC 2025 Jun 13. (PMC12165438; doi:10.1146/annurev-devpsych-120621-042753)
Supplement: Supplemental table 2 [file NIHMS2084123-supplement-Supplemental_table_2.pdf]

A developmental social neuroscience perspective on infant autism intervention
Annual Review of Developmental Psychology
Geraldine Dawson, PhD, Amber D. Rieder, PhD, and Mark H. Johnson, PhD

Supplemental Table 2. Studies of autism interventions for infants at or below 12 months of age

Study	Age initiated	Population	Length of intervention	Therapeutic approach	Fidelity assessed	Domains targeted	Child-related outcome measures	Results
(Green et al 2002)	14 months	Infant sibling with early behavioral signs (n=1)	3 years, 25-36 hr/week at home	Intensive ABA one-on-one with therapist	Yes	Infant learning, social, and communication skills	At 36 and 60 months, standardized tests of cognitive, communication, and adaptive skills completed by professionals who are not involved in intervention.	At 3-5 years of age, the child performed above her chronological age level on norm-referenced tests of cognitive and language skills and in a regular public school kindergarten classroom.
(Vismara 2008)	9 months	Infant siblings with early behavioral signs (n=1)	3 months, 12 sessions, 1.5 hr each, at home	Infant adapted Early Start Denver Model caregiver-coaching	Yes	Infant attention, motivation to engage, dyadic engagement, verbal/nonverbal communication, imitation, joint attention	At 3 month and 24 month follow up, observations of utterances and imitative behaviors, standardized tests of cognitive and communication skills, Autism Diagnostic Observation Schedule (ADOS)	Caregivers acquired intervention techniques. At 12 months, child's spontaneous utterances, social initiation, and imitative behaviors increased with parent and therapist. At 18-24 months, child's autism-related behaviors had declined.
(Green et al 2013)	8–10 months	Infant siblings who received intervention (n=7) compared to infant siblings (n=37) and lower likelihood	5-months, 12 sessions, 1.5 hr each at home	Video-aided and interaction-focused caregiver-coaching	Trained therapists	Caregiver responsiveness to and synchrony with infant	At 14-15 months, observations of caregiver–infant Interaction: standardized tests of expressive and receptive language, visual reception, gross and fine motor skills; Autism Observation Scale for Infants (AOSI); gap-overlap task	Intervention was feasible and acceptable to caregivers. Caregivers' sensitive responding and non-directiveness increased compared to both comparison groups. There was no consistent change on AOSI, standardized tests, or gap-overlap task.

Supplemental Material: Annu. Rev. Dev. Psychol. 2023. 5:89-113
https://doi.org/10.1146/annurev-devpsych-120621-042753

		infants (n=33) who did not receive intervention.						
(Steiner et al 2013)	12 months	Infant sibling with autism behavioral signs (n=3)	3 months, 10 sessions, 1 hr each at clinic and home	Pivotal response treatment caregiver-coaching; 8 sessions in clinic and 2 at home	Yes	Increasing infant motivation to engage in social interaction, frequency of communication attempts	At 15 months, observations of frequency of functional communication: eye contact and gesture with examiner or parent, standardized testing, and ADOS.	Caregivers acquired intervention skills. Children showed increased functional communication, including use of gesture; 2 of 3 children performed in average range on standardized tests and did not receive an autism diagnosis on the ADOS.
(Koegel et al 2014)	4, 7, and 9 months respectively	Infants referred for concerns about social engagement (n=3)	6, 12, or 4 weeks, 1 hr/week at home	Modified Pivotal Response Treatment, parent-education using practice-with-feedback	Yes	Increasing infant motivation to engage in social interaction	During intervention, observations of infant response to name, eye contact, and affect	All infants showed an increase in social engagement.
(Rogers et al 2014)	6-15 months	Infant siblings (n=4) or referred for concerns about social development (n=3) compared to infant siblings	12-weeks, 1 hr/week in clinic with 6-week maintenance period 1-hr clinic visits post-treatment weeks 2, 4, and 6	Infant adapted ESDM caregiver coaching model	Yes	Increasing parental sensitivity and responsivity to infant cues and infant attention, communication, affect, turn-taking, vocalizations, social interest and	At 15,18, 24, and 36 months, standardized testing, AOSI, ADOS, best estimate diagnosis	Caregivers mastered intervention skills. On standardized tests, language developmental rates of intervention group accelerated more steeply than other groups, moving into the average range by 24–36 months. At 36 months, intervention group had much lower

		(n=126) and lower likelihood infants				engagement, flexible play with toys		rates of autism diagnosis and cognitive delay.
(Baranek et al 2015)	12 months	Elevated autism signs on the First Year Inventory randomized to receive intervention (n=11) vs referred to community services (n=5)	6-8 months, 2 1-hr sessions/week for 6 weeks with 2 sessions over next 18 weeks at home	Adapted Responsive Teaching is relationship-focused caregiver coaching intervention aimed at increasing caregiver responsiveness	Yes	Social-communication and sensory regulation (arousal, attention, adaptability, toy play)	At 14, 22, and 32 months, standardized testing, adaptive behavior, sensory processing, joint attention	Intervention group showed improved child receptive language, socialization, and sensory hypo-responsiveness, and caregivers showed a less directive interactive style.
(Green et al 2015)	9 months (n=54)	Infant siblings randomized to intervention (n=28) or no intervention (n=26)	5 months, 12 1-hr sessions at clinic	Video Interaction to Promote Positive Parenting involving videotaping caregiver-infant interactions in home session and using videos for reflection and feedback regarding infant communication	Yes	Infant attentiveness to caregiver and caregiver understanding of infant communication and sensitive responding and emotional attunement	At 15-month intervention endpoint, AOSI, observations of infant attentiveness to the caregiver during interaction, quality of caregiver-child interaction, autism-related behaviors, standardized tests of language, ERPs to speech sounds, attention disengagement, and adaptive behavior	In the intervention group, infants showed increased infant attentiveness to caregiver, lower levels of autism-related behaviors, improved attentional disengagement and increased adaptive social behavior, and caregivers showed increased nondirectiveness. Intervention group also exhibited reduced ERP response to speech sounds and lower scores on standardized test of receptive language.
(Green et al 2017)	9 months	Infant siblings randomized to	5 months, 12	Video Interaction to Promote	Yes	Infant attentiveness to caregiver and caregiver	27 and 39 months – autism-related behaviors (blind-rated	Intervention group showed improvements in parent

		intervention (n=28) or no intervention (n=26)	1-hr sessions at clinic	Positive Parenting involving videotaping caregiver-infant interactions in home session and using videos for reflection and feedback regarding infant communication		understanding of infant communication and sensitive responding and emotional attunement	AOSI or ADOS), observations of caregiver–child social interaction, standardized measures of cognitive, motor, language abilities, adaptive behavior, clinical diagnosis (ADOS at 39 months)	nondirectiveness and synchrony, and children showed increased attentiveness/communication initiation and a reduction of autism-related behaviors measured over time. At 39 months, there was no effect of the intervention on categorical diagnostic outcome or standardized tests of language.
(Watson et al 2017)	13-16 months	Elevated autism signs randomized to intervention (n=45) vs referred to community services (n=42)	8 months, 30 sessions and 6 additional contacts, at home	Adapted Responsive Teaching is relationship-focused caregiver coaching intervention aimed at increasing caregiver responsiveness	Yes	Social-communication and sensory regulation (arousal, attention, adaptability, toy play)	At 9 months after onset of intervention, communication and symbolic behavior scales, sensory processing assessment, standardized tests of cognitive and language ability, adaptive behavior, diagnosis (ADOS)	In the intervention group, caregivers showed increased responsiveness. No overall effect of intervention on child outcomes, including social communication and sensory-regulatory behaviors. Caregiver responsiveness was related to improvements in children's communication and sensory regulation.
(Jones et al 2017)	9-11 months with follow up at 12 and	Infant siblings randomized to intervention (n=19) or assessment	10 weekly 60–85-minute sessions at home	Prompting First Relationships is a caregiver coaching intervention	Yes	Social attention, parental responsivity to infant social communicative cues	At 12 and 18 months, habituation to faces and objects was used to measure sustained attention and learning speed. EEG theta power to social and nonsocial videos was	Intervention groups showed faster habituation times to faces, greater increase in frontal EEG theta power, and P400 response to faces

	18 months.	and monitoring (n=14) and a lower likelihood comparison group of infants		using video, role-playing, and reflective practice designed to promote infant contingent responding, positive affect, self-regulation, and caregiver responsivity to infant communicative cues.		and infant contingent responding	measured as an index of attention engagement, and event-related potentials (ERPs) to faces and objects were measured as an index of the speed and depth of processing of social and non-social stimuli.	comparable to age-matched low-likelihood participants.
(Whitehouse et al 2019)	12 months	Infants exhibited ≤ 3 autism-related behaviors randomized to intervention (n=50) or treatment as usual (n=53)	5 months, 10 sessions at home	Video Interaction to Promote Positive Parenting involving videotaping caregiver-infant interactions in home session and using videos for reflection and feedback	Yes	Infant attentiveness to caregiver and caregiver understanding of infant communication and sensitive responding and emotional attunement	Post-intervention, AOSI, autism-related behaviors, observations of the quality of caregiver-infant interaction, standardized tests of cognitive and language ability, adaptive behavior	Intervention group did not differ from non-intervention group in terms of autism-related behaviors, quality of caregiver-infant interaction, infant attentiveness, caregiver nondirectiveness and responsiveness, standardized measures of cognitive and language ability, and adaptive behavior. Infants in intervention group had lower positive affect and higher caregiver-reported language skills.

				regarding infant communication				
(Whitehouse et al 2021)	12 months	Infants exhibited ≤ 3 autism-related behaviors randomized to intervention (n=50) or treatment as usual (n=53)	5 months, 10 sessions at home	Video Interaction to Promote Positive Parenting involving videotaping caregiver-infant interactions in home session and using videos for reflection and feedback regarding infant communication	Yes	Infant attentiveness to caregiver and caregiver understanding of infant communication and sensitive responding and emotional attunement	At 18, 24, and 36 months, AOSI, ADOS, autism-related behaviors, observations of the quality of caregiver-infant interaction, standardized tests of cognitive and language ability, adaptive behavior	Intervention group showed reduction in autism-related behaviors when measured across time points, lower odds of meeting criteria for autism diagnosis, improved caregiver-reported and communication skills. Caregiver sensitive responsiveness attenuated by 24 months post-intervention.

REFERENCES

Baranek GT, Watson LR, Turner-Brown L, Field SH, Crais ER, et al. 2015. Preliminary efficacy of adapted responsive teaching for infants at risk of autism spectrum disorder in a community sample. *Autism Res Treat* 2015: 386951

Green G, Brennan LC, Fein D. 2002. Intensive behavioral treatment for a toddler at high risk for autism. *Behavior modification* 26: 69-102

Green J, Charman T, Pickles A, Wan MW, Elsabbagh M, et al. 2015. Parent-mediated intervention versus no intervention for infants at high risk of autism: a parallel, single-blind, randomised trial. *Lancet Psychiatry* 2: 133-40

Green J, Pickles A, Pasco G, Bedford R, Wan MW, et al. 2017. Randomised trial of a parent-mediated intervention for infants at high risk for autism: longitudinal outcomes to age 3 years. *J Child Psychol Psychiatry* 58: 1330-40

Green J, Wan MW, Guiraud J, Holsgrove S, McNally J, et al. 2013. Intervention for infants at risk of developing autism: a case series. *J Autism Dev Disord* 43: 2502-14

Jones EJH, Dawson G, Kelly J, Estes A, Webb SJ. 2017. Parent-delivered early intervention in infants at risk for ASD: Effects on electrophysiological and habituation measures of social attention. *Autism Res* 10: 961-72

Koegel L, Singh A, Koegel R, Hollingsworth J, Bradshaw J. 2014. Assessing and Improving Early Social Engagement in Infants. *J Posit Behav Interv* 16: 69-80

Rogers SJ, Vismara L, Wagner AL, McCormick C, Young G, Ozonoff S. 2014. Autism treatment in the first year of life: a pilot study of infant start, a parent-implemented intervention for symptomatic infants. *J Autism Dev Disord* 44: 2981-95

Steiner AM, Gengoux GW, Klin A, Chawarska K. 2013. Pivotal response treatment for infants at-risk for autism spectrum disorders: a pilot study. *J Autism Dev Disord* 43: 91-102

Vismara LA,Rogers S. 2008. The Early Start Denver Model: A Case Study of an Innovative Practice. *Journal of Early Intervention* 31: 91-108

Watson LR, Crais ER, Baranek GT, Turner-Brown L, Sideris J, et al. 2017. Parent-Mediated Intervention for One-Year-Olds Screened as At-Risk for Autism Spectrum Disorder: A Randomized Controlled Trial. *J Autism Dev Disord* 47: 3520-40

Whitehouse AJO, Varcin KJ, Alvares GA, Barbaro J, Bent C, et al. 2019. Pre-emptive intervention versus treatment as usual for infants showing early behavioural risk signs of autism spectrum disorder: a single-blind, randomised controlled trial. *Lancet Child Adolesc Health* 3: 605-15

Whitehouse AJO, Varcin KJ, Pillar S, Billingham W, Alvares GA, et al. 2021. Effect of Preemptive Intervention on Developmental Outcomes Among Infants Showing Early Signs of Autism: A Randomized Clinical Trial of Outcomes to Diagnosis. *JAMA Pediatr* 175: e213298
